# Role of pattern recognition receptors in sensing *Mycobacterium tuberculosis*

**DOI:** 10.1016/j.heliyon.2023.e20636

**Published:** 2023-10-04

**Authors:** S.M. Neamul Kabir Zihad, Nazifa Sifat, Mohammad Ashraful Islam, A.S.M. Monjur-Al-Hossain, K.M. Yasif Kayes Sikdar, Md Moklesur Rahman Sarker, Jamil A. Shilpi, Shaikh Jamal Uddin

**Affiliations:** a Department of Pharmacy, State University of Bangladesh, Dhaka, 1205, Bangladesh; b Department of Pharmacy, ASA University of Bangladesh, Dhaka, 1207, Bangladesh; c Department of Pharmacy, University of Asia Pacific, Dhaka, 1205, Bangladesh; d Department of Pharmaceutical Technology, University of Dhaka, Dhaka, 1000, Bangladesh; e Pharmacy Discipline, Life Science School, Khulna University, Khulna, 9208, Bangladesh; f Department of Pharmacy, Gono University, Nolam, Mirzanagar, Savar, Dhaka 1344, Bangladesh

**Keywords:** *Mycobacterium tuberculosis*, Pattern recognition receptor, Cytokines, Phagocytosis, Inflammasome

## Abstract

*Mycobacterium tuberculosis* is one of the major invasive intracellular pathogens causing most deaths by a single infectious agent. The interaction between host immune cells and this pathogen is the focal point of the disease, Tuberculosis. Host immune cells not only mount the protective action against this pathogen but also serve as the primary niche for growth. Thus, recognition of this pathogen by host immune cells and following signaling cascades are key dictators of the disease state. Immune cells, mainly belonging to myeloid cell lineage, recognize a wide variety of *Mycobacterium tuberculosis* ligands ranging from carbohydrate and lipids to proteins to nucleic acids by different membrane-bound and soluble pattern recognition receptors. Simultaneous interaction between different host receptors and pathogen ligands leads to immune-inflammatory response as well as contributes to virulence. This review summarizes the contribution of pattern recognition receptors of host immune cells in recognizing *Mycobacterium tuberculosis* and subsequent initiation of signaling pathways to provide the molecular insight of the specific Mtb ligands interacting with specific PRR, key adaptor molecules of the downstream signaling pathways and the resultant effector functions which will aid in identifying novel drug targets, and developing novel drugs and adjuvants.

## 1 Introduction

The dynamic interaction between any pathogen and the human immune system determines the outcome of the disease. In the case of *Mycobacterium tuberculosis* (Mtb), there could be several outcomes depending upon the manner of the interaction. Mtb enters the human body either through inhalation of small droplets (≥2 μm) in the lower respiratory tract or through ingestion of infected matters in the gut. As it is attached with different types of immune cells a cross-play between Mtb and the cells takes place [1,2]. The effective initial immune response can result in the successful eradication of the infection. Mtb can evade the immune response through different virulence factors and cause primary tuberculosis (TB). The pathogen can also trigger a non-sterile response and become latent that can progress to active TB in the later lifespan of the patient [1]. The immunopathology of TB is very interesting in nature. The initial immune response involves the phagocytotic engulfment of the pathogen and initiation of an inflammatory immune response due to the release of two major elements, cytokines and chemokines, by the phagocytes followed by granuloma formation and recruitment of immune cells [3]. This response is important for both the host and the pathogen itself. For the host, this response can further proceed to the shrinkage of granuloma leaving calcification and make the pathogen susceptible to T lymphocytes. But, Mtb has evolved itself so successfully that instead of calcification it can promote cavitation i.e. necrotic cell death and leakage of granuloma content through which it can spread to new hosts [2].

Cells of the myeloid lineage including monocyte, macrophage, dendritic cell, and neutrophil along with natural killer cells mount the initial response against Mtb since these cells are the primary niches of Mtb [4,5]. The response is initiated by a variety of pattern recognition receptors (PRR) found on the host immune cells recognizing pathogen-associated molecular patterns (PAMP) expressed by Mtb followed by either opsonical or non-opsonical internalization of the pathogen [6]. Afterward, numerous biochemical events specific to the receptor type take place and end through effector functions to eradicate the pathogen including phagocytosis, oxidative burst, phagosomal acidification, autophagy, apoptosis, pyroptosis, and inflammasome activation [7].

In innate immune cells, different types of PRRs are found to be involved in the recognition of Mtb PAMPs by phagocytes including toll-like receptors (TLRs), nucleotide-binding oligomerization domain (NOD)-like receptors (NLRs), C-type lectine receptors (CLRs), complement receptors (CRs), scavenger receptors (SRs), absent in melanoma receptor-2 (AIM2), aryl hydrocarbon receptor (AhR), and CD14 receptors [[8], [9], [10], [11]]. Mtb cell wall is composed with a complex array of molecular patterns specific to mycobacteria including lipoarabinomannan (LAM), mannose capped lipoarabinomannan (ManLAM), lipomannan (LM), and phosphatidylinositol mannoside (PIM). Furthermore, secreted effector proteins and nucleic acid from Mtb function as PAMP [12]. Different PRRs recognize the pathogen by interacting with specific PAMP and this interaction determines the ultimate fate of the pathogen as the pathogen itself uses several PAMPs to modulate the immune response to ensure its survival. The present review was undertaken to accumulate, organize and represent all the updated information regarding the interaction of different PRRs with respective Mtb PAMPs and associated signaling cascades. We mainly focused on the role of these PRRs in containing the disease by sensing the causative organism to provide the molecular insight of the specific Mtb ligands interacting with specific PRR, key adaptor molecules of the downstream signaling pathways and the resultant effector functions which will aid in identifying novel drug targets, and developing novel drugs and adjuvants.

### 1.1 Toll-like receptors (TLRs)

**Fig. 1:**
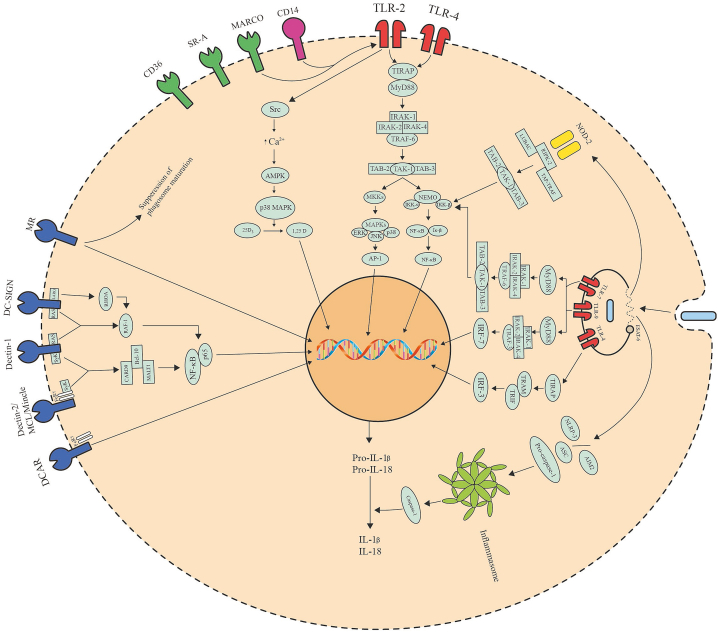
Major immune signaling pathways following recognition of Mtb PAMPs by host PRRs.

TLRs are a group of *trans*-membrane proteins that are mostly found on the surface of dendritic cells and macrophages, and serve as the key recognition molecules in the innate immune response against Mtb [13]. Of the 12 members of the TLR family, TLR2 has been shown to play an active role in conjunction with TLR1 and TLR6, TLR4 and TLR9 in recognizing Mtb PAPMs and initiating immune response (Table 1) [[14], [15], [16]]. Stimulation of these TLRs by Mtb antigens results in complex intracellular signaling cascades that involve the recruitment of TIR domain-containing adaptor (TIRAP-Mal) and myeloid differentiation primary response protein 88 (MyD88), the key adaptor molecules of this process, which is followed by gathering of IL-1 receptor-associated kinases (IRAK), TNF receptor-associated factor-6 (TRAF-6), TGF-β activated protein kinase-1 (TAK1) along with its binding proteins TAB2 and TAB3, that leads to the activation of nuclear transcription factor-κB (NF-κB) essential modulator (NEMO). NEMO recruits IκB kinases (IKK-α and IKK-β) leading to phosphorylation of IκB, nuclear translocation of NF-κB. In parallel, recruitment of TAK1 leads to activation of mitogen-activated protein kinase (MAPK) kinases (MKKs), activation of different MAPKs (extracellular signal-regulated kinase (ERK) 1/2, c-Jun N-terminal kinase (JNK) and p38), and translocation of transcription activator protein-1 (AP-1) (Fig. 1). Finally, this pathway ends in the expression or production of pro-inflammatory cytokines like IL-1, IL-6, IL-8, IL-12, TNF-α, type-I IFNs, and NO **(**Table 1**)** [17]. Secretion of TNF-α, T-cell polarizing IL-12, and following IFN-γ secretion by helper T cell leads to several protective mechanisms against Mtb including the generation of reactive nitrogen and oxygen species, phagolysosome fusion and acidification, and autophagy [[18], [19], [20]]. MyD88 is an integral part of the innate response towards Mtb as mice deficient in MyD88 show higher susceptibility to Mtb than wild type [21].

**Table 1:** Host immune receptors interacting with Mtb ligands and the consequences.

PRR class	Receptor name	Mtb ligand	Secreted cytokines	Functions
Toll-like receptor	TLR2	di-(TLR2/TLR6) or tri-(TLR2/TLR1) acylated lipoproteins, 19-kDa lipoprotein, LprA, LprG, LprE, rLrp, 30-kDa antigen, 38-kDa antigen, MymA, PPE18, PPE-57, heat shock protein 60, MTP83, Rv1509, Rv2659c, Rv1738, Rv2627c, Rv2628, ESAT-6, LAM, LM, ManLAM, and PIM.	IL-1β, IL-2, IL-4, IL-6, IL-8, IL-10, IL-12, IL-22, TNF-α, TGF-β, MCP-1, and IFN-γ.	Generation of RNS and ROS, apoptosis, T-cell priming, induction of Th cell responses, and evoking immunological memory response.
	TLR3	dsRNA and tRNA.	IL-10 and IL-12.	Boosting TLR8 signaling.
	TLR4	Heat shock proteins, 38-kDa antigen, RpfE, Rv0652, Rv0335c, Rv2659c, Rv1738, Rv2627c, Rv2628, GrpE, and HBHA.	IL-1β, IL-2, IL-6, IL-8, IL-10, IL-12, IL-17, IL-23, TNF-α and IFN-γ.	Dendritic cell activation, MHC I and II antigen processing, boosting Th1 and Th17 mediated response, autophagy, and apoptosis.
	TLR7	ssRNA	IFN-α and IFN-β.	Autophagy
	TLR8	tRNA and phagosomal RNA	IL-12, IL-18 and IFN-γ.	Autophagy
	TLR9	dsDNA	TNF-α, IFN-α and IFN-β.	Induction of Th1 response And MHC-I-Ag cross-processing.
NOD-like receptor	NOD2	Muramyl dipeptide	IL-1β, IL-6, IL-8, IL-10, IL-12, TNF-α, CXCL-2, CCL-5, and MCP-1.	Generation of RNS, autophagy, potentiation of TLRs.
	NPLR3	dsDNA	IL-1β	Inflammasome activation
C-type lectin receptor	Dectin-1	Unknown	IL-1β, IL-2, IL-10, IL-12, IL-17, IL-23, TNF-α, IFN-γ, and CXCL2.	Phagocytosis, dendritic cell maturation, respiratory burst, and production boosting Th1 and Th17 mediated response.
	Dectin-2	ManLAM	IL-1, IL-2, IL-6, IL-12, IL-17, TNF-α, IFN-γ and MIP-2.	DC maturation and boosting Th17.
	Mincle	TDM, TMM	IL-1α, IL-1β, IL-6, IL-8, IL-10, TNF-α, and G-CSF.	Autophagy, production of NO and ROS, delaying phagosome maturation and stimulating surface CD11b expression.
	MCL	TDM	IL-1β, IL-6, MIP-2 and TNF-α	Phagocytosis, inhibition of NLRP3 inflammasome activation, respiratory burst, and stimulating the expression of Mincle.
	DCAR	PIMs	IL-12 and IFN-γ.	Stimulating Th1 response.
	CR3	LAM and PIM		Opsonic and non-opsonic phagocytosis.
	DC-SIGN	LAM, ManLAM, PIM, α-glucan, 19 kDa, 38 kDa and 45 kDa antigens.	IL-6, IL-10, IL-12, and CXCL-8.	Th1 and Th17 response inhibition.
	MR	ManLAM, PIM, LM, 38‐kDa glycoprotein, 19-kDa antigen, and other mannosylated proteins		Phagocytosis, inhibition of phagolysosome fusion, and inhibition of Th17 response.
	DCIR	Unknown		Inhibition of TLR signaling.
	SP-A	ManLam, LM, 60-kDa glycoprotein and glycoprotein Apa.	IL-10 and TGF-β1	Agglutination, phagocytosis, induction of MR expression, and suppression of NO and ROS.
	SP-D	LAM, LM and PILAM		Inhibition of phagocytosis and enhancing phagolysosome fusion.
	MBL	ManLAM		Complement activation.
Scavenger receptors	SR-A	TDM	IL-4 and TNF-α.	Phagocytosis and suppression of Th1 response.
	MARCO	TDM	IL-1β, IL-6, and TNF-α.	Phagocytosis, autophagy, and production of ROS and RNS.
	SR-B1	ESAT-6		Transcytosis of Mtb across microfold cells.
	CD36	ManLAM and LM	TNF-α	Suppression of NO and promoting lysosomal dysfunction.
	CD163			Disease biomarker.
	AIM			Production of ROS and antimicrobial, and autophagy.
Absent in Melanoma receptor	AIM2	ssDNA	IL-1β and IL-18.	Inflammasome activation, autophagy, and boosting Th1 response.
Aryl hydrocarbon receptor (AhR)		Naphthoquinone phthiocol		Inhibition of phagocytosis.
CD14		Chaperonin 60.1	IL-1β, TNF-α, IL-6, IL-8, and IL-12	Phagocytosis and facilitating signaling of TLR2 and MARCO.
Triggering receptor expressed on myeloid cells 2 (TREM-2)				Proinflammatory Th1 response
Macrophage galactose-type lectin (MGL)-1				T-cell mediated control of Mtb
Retinoic acid inducible gene I (Rig-I)		RNA	IFN-β	
Galectins	Galectin-3			Autophagy
	Galectin-8			Autophagy
	Galectin-9			Autophagy

TLR2 is responsible for the recognition of Mtb cell wall antigens like di-(TLR2/TLR6) or tri-(TLR2/TLR1) acylated lipoproteins, 19-kDa lipoprotein, LprA lipoprotein, LprG lipoprotein, 30-kDa antigen, 38-kDa antigen, MymA, proline-proline-glutamic acid (PPE)-57, LAM, LM, and PIM; and initiate an innate immune response in macrophages and dendritic cells [14,[22], [23], [24], [25], [26]]. Ligation of TLR2 with Mtb ligands leads to the generation of IL-1β, IL-2, IL-8, IL-12, IL-22, TNF-α, MCP-1, and IFN-γ through classical MyD88/NK-κB and MAPK pathway, generation of RNS and ROS, and apoptosis **(**Table 1**)** [[25], [26], [27], [28], [29], [30]]. A recent study shows that Mtb LAM challenged neutrophils to generate IL-6 and IL-8 via MyD88/MAPK independent TLR2/1 mechanism [31]. Yang and colleagues reported about protein kinase C zeta (PKCzeta) that it has a crucial effect in 19-kD antigen-TLR2 mediated generation of ROS as well as inflammatory cytokines (TNF-α and IL-6) from macrophage by upregulating ERK 1/2 where macrophage surface protein CD157 aids in the compartmentalization of TLR2 and PKCzeta [32]. Another mechanism involves the participation of TLR2/1 when induced by Mtb lipoprotein Lpq-H or 19-kDa lipoprotein that leads to phosphorylation of Src kinase and subsequent intracellular Ca^2+^ influx. This pathway further proceeds through phosphorylation of AMPK-α and activation of p38-MAPK. These events result in the conversion of vitamin-D pro-hormone 25D3 to 1,25D3 by up-regulating Cyp27b1 hydroxylase. Upon translocation into the nucleus by the vitamin-D receptor, 1,25D activates the transcription of antimicrobial peptide cathelicidin and subsequent autophagy (Fig. 1) [33]. Another Mtb lipoprotein MTP83 induces apoptosis in infected macrophages via TLR2/p38/COX-2/PGE_2_ pathway [34]. Mtb secreted protein ESAT-6 stimulates the production of IL-6 as well as TGF-β by TLR2/MyD88 dependent manner and induces helper T cell responses, ROS generation, and apoptosis [35,36]. Though it has been reported that TLR2 mediated signaling can prime T-cell response, it is not the sole mechanism [25,37]. Mtb itself utilizes TLR2 in modifying the host environment to ensure its survival. It has been seen that TLR2 dependent ERK 1/2 plays an important role to increase anti-inflammatory IL-10 secretion, suppression of IL-12, and T-cell priming [38]. Lipoprotein, LprE from Mtb cell wall downregulates p38-MAPK and TLR2 mediated autophagy. It also decreases phagolysosome fusion by downregulating the transcription of IL-12 and IL-22 [39]. Mtb effector protein ESAT-6 is responsible for phagosomal upregulation of IL-10 and suppression of IL-12 [40]. Mtb also secretes other proteins namely, PPE18 and heat shock protein 60, that induces IL-10 secretion via TLR2/MyD88/p38 MAPK mechanism [41,42]. The rLrp of Mtb inhibits the generation of pro-inflammatory cytokines, especially IL-12 as well as TNF-α, and downregulates antigen presentation by MHC-II through ligation with TLR2 and subsequent PI3K/AKT pathway [43]. An interesting immunoinhibition by Mtb involves ligation of ManLAM and TLR2 on B cells that leads to activate MyD88 dependent AP-1 and NF-κB. As a results increases the production of IL-10, inhibition of IFN-γ, and increased production of IL-4 [44]. A recent study illustrates that Mtb signature protein Rv1509 initiates its immunomodulatory function through TLR2 receptor resulting in dendritic cell maturation, decreased expression of DC-SIGN, and evoked immunological memory response [45]. Through the balancing between pro and anti-inflammatory signaling by TLR2 determines its role in Mtb clearance. It is for sure that TLR2 is an integral part of anti-Mtb immunity since TLR2 deficient mice have been shown to have diminished immune-inflammatory response [46]. Furthermore, polymorphisms in the TLR2 gene are reported to impair the immune response towards Mtb and increase the risk of pulmonary tuberculosis [47,48].

Recognition and signaling through TLR4 can be initiated by heat shock proteins, 38-kDa antigen, resuscitation‐promoting factor E (RpfE), Mtb ribosomal protein Rv0652, and heat shock protein co-factor GrpE [[49], [50], [51], [52], [53]]. Ligation of TLR4 with Mtb ligands results in the production of IL-1β, IL-2, IL-6, IL-12, IL-17, IL-23, TNF-α as well as IFN-γ through MyD88/NK-κB and MAPK [51,54]. TLR4 activation is very crucial for dendritic cell activation, MHC I and II antigen processing, boosting Th1 and Th17 mediated response, and autophagy **(**Table 1**)** [53,54]. Another study suggests that activation of both TLR2 and TLR4 by Mtb 38-kDa antigen can lead to ER stress-induced apoptosis through TLR-MAPK-dependent signaling pathway and production of ROS [55]. Beside MyD88 signaling, TLR4 can initiate signaling pathway through the TIR domain-containing adapter protein (TIRAP), TIR domain-containing adapter protein inducing IFN-β (TRIF), TRIF-related adapter molecule (TRAM), as well as TNF receptor-associated factor (TRAF)-3 that results from interferon regulatory factor (IRF)-3 downstream and leads to the production of IFN-β (Fig. 1) [17]. Jang et al. reported that Mtb ESAT-6 is a major contributor to the virulence of Mtb that utilizes this pathway [56]. The heparin-binding hemagglutinin (HBHA) of Mtb that serves as a diagnostic parameter of tuberculosis disease imparts strong immunostimulatory effect through TLR4-dependent signaling resulting in DC maturation, activation of simple T cells, the polarization of T cells in order to produce IFN-γ, as well as subsequent cytotoxic T cell-mediated toxicity [57]. TLR4 activation augments the DNA-dependent cytosolic surveillance by Mtb through enhancing the production of IFN-β helping in long-term disease persistence [58]. Zhang and colleagues reported an interesting role of TLR4 in negative immunomodulation. TLR4 activation in Mtb challenged macrophages leads to the upregulation of miR-32–5p, a micro RNA that attenuates expression of IL-1β, IL-6, as well as TNF-α by targeting follistatin-like protein 1 (FSTL1), an endogenous mediator of inflammation that activates JNK, ERK 1/2, and NF-κB [59]. Another Mtb protein PE6 (Rv0335c) interacts with TLR4 of macrophage cell surface and evokes a canonical NK-κB mediated inflammatory response that leads to the secretion of TNF-α, IL-6, and IL-12. These events induce unfolded protein response in endoplasmic reticulum leading to apoptotic cell death. However, this protein, PE6 has also been found to suppresses autophagy, the major innate response [60]. Despite negative immunomodulation, TLR4 plays a pivotal role in inhibiting local growth as well as inducing immune clearance of Mtb [52]. Little work has been done regarding the association between TLR4 gene polymorphism and TB risk, and the results show a positive relationship [61,62].

TLR9 is situated on the membrane of phagosome and it can sense the unmethylated CpG dinucleotides in the bacterial DNA [63]. Poecheim and colleagues have shown that activation of TLR9 by Mtb antigen encoding plasmid DNA increases pro-inflammatory cytokine (TNF-α) release in mice (Table 1) [64]. In addition, it is reported that TLR9 signaling co-operates with TLR2 signaling in mediating Th1 response against Mtb in mice by producing IL-12 via MyD88/NF- κB pathway [65]. Defect in TLR9 signaling results in disease persistence, though virulent Mtb strain has the capability to attenuate TLR9 signaling [66,67]. TLR9 has also been involved in MyD88 dependent production of type-I IFNs involving adaptor molecules IRAKs, TRAF-3, and IRF-7 (Fig. 1) resulting in MHC-I-Ag cross-presentation, and signaling through TLR2 can diminish this pathway by depleting IRAK-1 [68]. However, several human studies show a positive relation between TLR9 gene polymorphism and TB susceptibility marking its importance in anti-Mtb immunity [69,70].

Apart from these three TLRs, also other members of this receptor family can recognize Mtb products. Mtb dsRNA induces TLR3 activation that leads to produce regulatory IL-10 by the signaling pathway of MyD88/PI3K/AKT/NF-κB (Table 1) [71]. Endosomal TLR7 is activated by ssRNA of Mtb and shares the same pathway as TLR9 leading to MyD88/NF-κB activation and MyD88 dependent type-I IFNs production (Fig. 1) [68,72]. Bao and colleagues further explored the immunological role of TLR7 and they found that ligation of TLR7 with Mtb ssRNA induces the production of autophagy-related proteins leading to autophagosome formation [73]. Researchers have found upregulation of TLR8 expression in macrophages upon Mtb infection [74]. Keegan and colleagues showed that TLR8 of human macrophages can be activated by Mtb tRNA triggering the secretion of IL-18. Simultaneously, TLR8 and TLR3 on dendritic cells can sense Mtb tRNA, and synergistically produces bioactive IL-12 through the classical MyD88 dependent pathway. Afterward, IL-12 increases the level of IL-18 receptor on NK cells, facilitating IL-12 and IL-18 mediated induction of IFN-γ **(**Table 1**)** [75]. Charlotte et al. have postulated that TLR8 can sense the phagosomal Mtb RNA and observed increased recruitment of MyD88. Thay have showed that autophagy is the major effector function upon TLR8 medicated immune recognition of phagosomal Mtb RNA [76]. Direct association of TLR7 and TLR8 polymorphisms has been found with the increased susceptibility towards Mtb infection [69,77] but the mechanisms remain unknown. Several Mtb components are shown to interfere with general TLR signaling to impart immunity towards the pathogen from the human immune system. For example, Mtb secreted proteins, PtpA and Mce3E negatively regulate TLR signaling involving NF- κB and ERK-mediated cytokine production [78,79]. Mtb also suppresses the production of reactive oxygen and nitrogen species through the inhibition of TLR dependent activation of MAPKs by secreting proteins, AcpM and MptpB, respectively [80,81]. Another DNA binding protein of Mtb, EspR, directly binds with MyD88 and inhibits TLR dependent MyD88 signaling leading to reduced immune-inflammatory response and apoptosis, thus, supports Mtb survival within macrophage [82].

### 1.2 NOD-like receptors (NLRs)

NLRs, belonging to an intra-cellularly expressed cytoplasmic receptor family, can recognize microbial products and PAMPs including cell wall components and toxins [83]. Among approximately 20 members of this family NOD1, NOD2, NLRC4, NLRP3, and NLRP10 have been extensively researched and biologically described, and they are involved in mounting innate immune inflammatory response resulting in NF-κB activation, autophagy, and inflammasome activation as well as stimulating adaptive response against the virus, bacterial, and several other inflammatory conditions [[83], [84], [85], [86], [87], [88], [89]]. Mtb has evolved itself to escape from phagosome into the cytoplasm through several virulence mechanisms like ESX-1 secretion system [90], thus it is inevitable that cytoplasmic PRRs like NLRs are responsible for recognizing and containing cytoplasmic Mtb load.

NOD1 and NOD2 are associated with the endosomal membrane, both recognizing bacterial peptidoglycan. The γ-D-glutamyl-*meso*-diaminopimelic acid is predominantly found in gram-negative types of bacteria which can be recognized by NOD1 while NOD2 can recognize the muramyl dipeptide component of peptidoglycan of most bacteria [91,92]. Both NOD1 and NOD2 have three types of functional domains called N-terminal caspase activation recruitment domain (CARD) that interacts with downstream adaptor proteins, NACHT domain, and C-terminal leucine-rich repeat (LRR) domain (recognition of peptidoglycan) [83]. Upon recognition of the respective ligands, these NODs recruit receptor-interacting serine/threonine kinase (RIPK2) through the interaction between CARD-CARD which is followed by the recruitment of inhibitor of apoptosis proteins (IAP) and TNF-receptor associated factors (TRAFs) [93]. RIPK2 is poly-ubiquitylated by these IAP-TRAF complexes and facilitates the recruitment of the linear ubiquitination assembly complex (LUBAC). Afterward, RIPK2 forms a complex with LUBAC and TAK1 binding protein complex (TAK1-TAB2/3) [94,95]. NF-κB and MAKP pathways are activated through this complex cascade (Fig. 1) and subsequent secretion of pro-inflammatory mediators (IL-6, IL-8, TNF, MCP-1, and CXCL-2) and defensins [96,97]. To date, the role of NOD1 in immunity towards Mtb is not clear, but one study showed that it potentiates the efficacy of NOD2 and other TLRs in cytokine production [98]. NOD2 is the most studied NLR among all. Gandotra and colleagues showed that NOD2 recognition is crucial for the generation of IL-12, TNF-α, chemotactic cytokine CCL5, and NOS from macrophages. They also showed that DCs produce IL-6 as well as IL-10 in NOD2 dependent manner **(**Table 1**)** [99]. In other studies with NOD2 deficient mice showed diminished cytokine including IL-1β and TNF-α production from infected alveolar macrophages than wild-type resulting in impaired resistance to Mtb infection [100,101]. A recent survival experiment with NOD2 deficient mice resulted in decrease survival rate irrespective of the presence or absence of other PRRs [102]. In addition to this, Juarez and colleagues found that NOD2 recognition results in recruitment of autophagy-related proteins to Mtb containing autophagosome stating its role in autophagy [103]. NOD2 also works synergistically with TLRs (e.g. TLR4) in improving dendritic cells efficacy and subsequent T call activation as well as reducing drug dose [104,105]. Human studies indicate that polymorphism in NOD2 is directly connected with increased Mtb susceptibility among the Chinese and Afro-American population [106,107].

Besides the production of inflammatory mediators, inflammasome activation against Mtb is another immune response implicated by NLRs, particularly pyrin domain-containing-3 (NLRP3). This protein is very important for NLRP3 inflammasome assembly, a key effector mechanism of innate immune response [83]. Though it is difficult to point the specific Mtb ligands of NLRP3, studies prove that it is activated by 6-kDa early secreted antigen (ESAT-6) secreted by Mtb by the ESX-1 system that mediates the formation of pores in the phagosome causing the Mtb to escape into the cytosol. This pathway proceeds when the adaptor molecule ASC (an apoptosis-associated speck-like protein containing a carboxy-terminal CARD) is recruited which in turn recruits pro-caspase-1 via the interaction between CARD-CARD leading to the formation of NLRP3 inflammasome assembly. This inflammasome assembly activates caspase-1 from pro-caspase-1 and it mediates the maturation of IL-1β from pro-IL-1β produced through TLR mediated signaling (Fig. 1) resulting in pyroptotic clearance of Mtb **(**Table 1**)** [108]. The activation of NLRP3 inflammasome and subsequent cleavage of pro-IL-1β requires a number of additional signals to commence. One established mechanism is cytosolic depletion in K^+^ caused by pore formation and membrane damage [109]. NIMA-related kinase (NEK)-7 has been identified to bind with NLRP3 and acts as an initiator of K^+^ depletion [110]. In addition, active cathepsin B from the damaged lysosomes also triggers NLRP3 assembly in cytosol [111]. Basu and colleagues found that dsRNA from Mtb can also trigger the above-mentioned pathway leading to NLRP3 inflammasome activation [112]. Recently a contradictory report has been published stating that NLRP3 knockdown, as well as its pharmacological inhibition, reduces Mtb growth in macrophages pointing to a favorable role of NLRP3 in Mtb survival [113]. Mtb has evolved itself to escape this effector function. A Mtb enzyme, Zn-metalloprotease reduces caspase-1 activity and inhibits the activation of NLRP3 inflammasome [114]. Furthermore, IFN-β produced from Mtb challenged myeloid cells inhibits IL-1β production by inducing IL-10 [115]. Yet, there is a significant association between polymorphisms in NLRP3 and clinical outcomes in TB patients [116]. Thus, NLRP3 inflammasome serves as an important sterilizing route against Mtb.

### 1.3 C-type lectin receptors (CLRs)

C-type lectin receptors or CLRs are a superfamily of immunoreceptors containing 17 groups of both soluble and *trans*-membrane proteins expressed by the cells which are situated on the myeloid lineage including macrophages, dendritic cells, monocytes, and neutrophils and they contain one or more carbohydrate-binding domains. CLRs act as PPR recognizing diverse range of microbial carbohydrates, lipids, proteins, and inorganic ligands in a Ca^2+^ dependent or independent manner [117]. CLRs are also involved in maintaining body homeostasis, cell-cell adhesion, and imparting immunity to autoimmune diseases, parasites, diabetes, atherosclerosis, arthritis, inflammatory conditions, and allergy [118]. Signaling motifs, immunoreceptor tyrosine-based activation motif (ITAM), and immunoreceptor tyrosine-based inhibitory motif (ITIM) are present in CLRs within either cytoplasmic tails or the signaling subunits associated with the CLRs, e.g. gamma chain of the Fc receptor (FcRγ) that dictate the type of downstream signal upon ligand ligation [119]. CLRs have been a key arsenal of the human immune system against tuberculosis with both membrane-bound and soluble CLRs being involved in host-pathogen interactions. Membrane-bound CLRs include dendritic cell-associated C-type lectins (Dectin-1, 2 and 3), macrophage-inducible C-type lectin (Mincle), macrophage C-type lectin (MCL), dendritic cell immune-activating receptor (DCAR), complement receptor, dendritic cell-specific ICAM-grabbing non-integrin (DC-SIGN), mannose receptor (MR) as well as dendritic cell immunoreceptor (DCIR), whereas soluble CLRs include surfactant protein A (SP-A), surfactant protein D (SP-D) and mannose-binding lectin (MBL) that can recognize different Mtb components as well as subsequently can trigger specific downstream signal [7,120]. Amongst these, ITAM containing CLRs are involved in the most established defense mechanism against Mtb. Upon ligand ligation with respective CLR, these ITAMs recruit syk kinase that subsequently induces a downstream signaling cascade activating caspase-recruitment domain protein 9–B cell lymphoma/leukemia 10–mucosa-associated lymphoid tissue lymphoma translocation protein 1 (CARD-9/BCL-10/MALT-1) complex through protein kinase C-δ (PKCδ). This pathway further proceeds via the activation of nuclear transcription actor-κB (NF-κB) essential modulator (NEMO) and nuclear factor-κB (NF-κB) p65-containing complexes, translocation of NF-κB (Fig. 1), and finally, expression of pro-inflammatory cytokines [118].

Dectin-1 contains a hemITAM motif and is involved in phagocytosis, dendritic cell maturation, respiratory burst, and production of IL-1β, IL-2, IL-12, IL-23, TNF-α, as well as CXCL2, and anti-inflammatory cytokine IL-10 (Table 1). Dectin-1 orchestrates adaptive immunity by stimulating the secretion of T-cell polarizing cytokines and disintegration of naive CD4^+^ T cells to Th1 or Th17 phenotype as well as, furthermore, stimulates helper T cells to secrete IL-17 and IFN-γ [121]. But this response is inhibited by MR and DC-SIGN [122]. Some evidences show that the anti-Mtb function of Dectin-1 sometimes requires simultaneous activation of TLR2 and TLR4 [123,124]. Apart from the classical syk-dependent pathway, Dectin-1 can activate NF-κB and subsequent cytokine production by the phosphorylation and activation of RAF1 by Ras proteins (Fig. 1) [121]. Though the specific Mtb ligand for Dectin-1 has not been discovered yet, this membrane-bound CLR is an integral part of anti-Mtb immunity since decreased expression of Dectin-1 resulted in reduced mycobacterial clearance in mice [125]. But, researchers have found no association between Dectin-1 and animal survival in KO mice [126].

Dectin-2 contains the FcRγ signaling axis having an ITAM motif and acts as a direct PRR for ManLAM of Mtb cell wall, thus, activates NF-κB and produces pro-inflammatory cytokines including IL-2, IL-6, IL-12, MIP-2, and TNF-α in a syk dependent manner. Interestingly, Dectin-2-ManLAM conjugation also results in the production of anti-inflammatory cytokine IL-10 [127,128]. Recognition of ManLAM by Dectin-2 results in DC maturation, Th17 polarizing through syk dependent pathway, and resultant secretion of IL-17 and IFN-γ (Table 1) [127,129].

Mincle and MCL also contain FcRγ-coupled ITAM motifs within the same gene cluster as Dectin-2 [127]. These two CLRs recognize the same Mtb component, trehalose-6,6′-dimycolate (TDM) or the cord factor, and triggers NF-κB mediated cytokine production [130,131]. Mincle has widely expressed on myeloid cells and B cells as well as upon ligand recognition, it triggers the syk dependent downstream signal to produce IL-1α, IL-1β, IL-6, IL-8, and G-CSF **(**Table 1**)** [132]. This pathway is a pre-requisite for TDM as well as its synthetic analogue, Trehalose-6,6-dibehenate (TDB) inducing Th1 and Th17 responses [133]. Intoduction of Th1 and Th17 responses by TDB also requires MyD88 dependent pathway through IL-1 signaling [134]. Though studies have been conducted to a lesser extent, trehalose monomycolate (TMM), which is a biosynthetic intermediate of TDM, shows potent binding affinity to Mincle and produces similar adjuvant efficacy [135]. Mincle receptor also works in association with activated TLR4 to induce autophagy in Mtb infected macrophages [136]. In addition, Mincle is essential for the production of nitric oxide (NO) and inflammation resolution in macrophages by suppressing NLRP3-dependent caspase-1 activation [137]. Negi and colleagues reported that human gut microbiota is crucial for Mincle expression on dendritic cells and anti-Mtb response [138]. Co-activation of Mincle and TLR-2 increases surface CD11b expression, ROS, and TNF-α production by neutrophils [139]. But it was later reported that integrin CD11b works as a negative regulator of the Mincle pathway to inhibit hyper-inflammation [140]. Patin and colleagues reported that Mincle-TDM conjugation can lead to the secretion of IL-10 and down-regulation of IL-12 secretion [141]. Another report from this lab shows that TDM can delay phagosome maturation by engaging with Mincle [142]. There is also evidence that Mincle is not essential for anti-Mtb immunity as responses were observed in Mincle deficient mice. In spite of these opposing findings, Mincle is considered a key component in anti-Mtb immunity since studies show associations between polymorphisms in the Mincle gene and TB susceptibility [143,144].

MCL is a newer member of the Dectin-2 family that arises from Mincle gene duplication and responsible for phagocytosis, respiratory burst, and syk-dependent cytokine production (Table 1) [131,145]. MyD88 mediated signaling is responsible for MCL and Mincle expression on macrophage [146]. Again, MCL expression is upregulated upon microbial challenge and mainly functions through stimulating the expression of Mincle and its signaling [147,148]. Studies show that MCL deficient mice show impaired secretion of IL-1β, IL-6, MIP-2, as well as TNF-α upon TDM challenge [131,149]. Polymorphism in the MCL gene is responsible for the increasing susceptibility to pulmonary TB [150]. But Zhang and colleagues reported that MCL inhibits NF-κB mediated transcription of pro-inflammatory cytokines and subsequent NLRP3 inflammasome activation [151].

DCAR is another receptor containing ITAM coupled FcRγ that recognizes mycobacterial PIMs and induces secretion of pro-inflammatory IL-12 and stimulates Th1 response by increasing IFN-γ (Table 1), but the mechanism is not fully understood [152].

Complement receptor 3, CR3 or CD11b, is upregulated upon Mtb infection and it recognizes mycobacterial LAM and PIM [153,154]. CR3 can induce both opsonic and non-opsonic phagocytosis in macrophages upon Mtb infection (Table 1) though they are not mandatory for host defense as an absence of CR3 does not alter the disease course in mice [155,156].

DC-SIGN is another *trans*-membrane CLR that recognizes Mtb ManLAM and its protective action requires prior activation of TLR signaling. DC-SIGN-ManLAM ligation recruits leukemia-associated RHO-GEF (LARG) and RHOA. These adaptors phosphorylate and activate the RAF1 signalosome resulting in phosphorylation as well as acetylation of the p65 subunit of NF-κB (Fig. 1), as a result it increases the pro-inflammatory responses i. e secretion of IL-6, IL-12, and CXCL-8 (Table 1) [118,157]. It also shares the same pathway for activation of RAF-1 by Dectin-1 (Fig. 1) [118]. Animal studies tell that it plays a vital role in anti-Mtb immunity [158,159]. It also recognizes LAM, PIM, α-glucan, 19 kDa, 38 kDa, and 45 kDa antigens of Mtb [160]. There are many contradictory findings regarding the function of DC-SIGN in TB pathogenesis. Gringhuis and colleagues reported that DC-SIGN exploits TLR signaling to increase anti-inflammatory IL-10 secretion [161]. Studies also show that DC-SIGN-ManLAM binding decreases LPS induced IL-12 production, Th1 response, Th17 response, and pro-inflammatory response in IL-4 activated macrophage [122,[162], [163], [164]]. Vitamin D supplementation boosts innate immune response by inhibiting DC-SIGN expression [165]. Polymorphism studies indicate both association and non-association of DC-SIGN gene polymorphism with TB susceptibility [[166], [167], [168], [169], [170]].

Mannose receptor (MR) recognizes a number of Mtb components including ManLAM, PIM, LM, 38‐kDa glycoprotein, 19-kDa antigen, and other mannosylated proteins [[171], [172], [173]]. In addition to its role as a marker in pulmonary tuberculosis, MR acts as an endocytic receptor promoting phagocytosis by macrophage upon ligand and inhibits fusion of Mtb containing phagosome with lysosome promoting Mtb growth by limiting PI(3)P generation [[172], [173], [174], [175]]. It also acts as the receptor for the Mtb-induced apoptotic cells for phagocytosis [176]. Ligand activated MR inhibits IL-12 production, promotes the secretion of anti-inflammatory cytokines by DC, and diminishes Th17 response (Table 1) [122,174]. Activation of PPARγ is linked to this anti-inflammatory function of MR to some extent [177].

DCIR is a rare ITIM motif coupled receptor that inhibits TLR mediated production of IL-12 and IFN-γ (Table 1), though the interacting Mtb ligand, as well as the mechanism, is still unidentified [178].

In addition to these membrane-bound CLRs, soluble lectins called collectins have also some part in TB pathology. Surfactant protein A and D are mainly secreted by the alveolar type 2 cells of the lung with SP-A recognizing Mtb ManLam, LM, 60-kDa glycoprotein, and glycoprotein Apa whereas SP-D recognizes LAM, LM, and PILAM [179,180]. SP-A serves as a key serum marker in TB, increases agglutination and phagocytotic uptake of Mtb by inducing MR expression (Table 1), but it suppresses the production of NO and ROS [179,181,182]. Samten and colleagues reported that SP‐A suppresses cell‐mediated immunity against Mtb by increasing the prodution of IL-10 as well as TGF-β1 [183]. Several reports suggest the association between TB risk and polymorphism in SP-A gene [[184], [185], [186], [187]]. On the other hand, in spite of inducing bacterial agglutination, SP-D inhibits phagocytotic uptake of Mtb by macrophage and limits Mtb growth by enhancing fusion of Mtb containing phagosome with the lysosome (Table 1) [180,188]. There have been reports of two SP-D gene polymorphisms and their association to TB susceptibility [189,190].

Another collectin, MBL recognizes ManLAM of Mtb cell wall and activates the lectin pathway of complement, thus, promotes clearance by phagocytosis **(**Table 1**)** [191]. Genetic studies confirm the anti-Mtb role of MBL since polymorphisms in the MBL gene confers to increased susceptibility [192,193].

### 1.4 Scavenger receptors (SRs)

Scavenger receptors are a super family of phagocytotic receptors expressed on macrophages and monocytes that recognize and internalize invasive pathogens by binding with common polyionic ligands, especially oxidized low-density lipoprotein. Though most SRs are found in membrane-bound forms some of them also exist in a soluble state. These receptors are involved in maintaining body homeostasis, antigen presentation, ROS production, apoptosis, angiogenesis, and pathogenesis of several diseases [194,195]. Among 8 classes of SRs, only two, class A and class B are engaged in immune-interaction with Mtb. Till now, type I and II class A SR (referred to as scavenger receptor A), macrophage receptor with collagenous structure (MARCO), scavenger receptor B1, and CD36 have been identified to be involved in recognizing and promoting internalization of Mtb (Table 1) [8,196].

Scavenger receptor A (SR-A) is a member of class A SRs capable of binding and internalizing microbial ligands as well as endogenous proteins. It is present in alveolar macrophages at a low level but is induced in foamy macrophages during chronic Mtb infection and mediate clearance of oxidized phospholipids [194,197]. TDM of Mtb acts as the ligand of SR-A, but their ligation seems to produce a very little effect as SR-A deficient mice were seen to have insignificant impairment in TNF-α production [9]. On the contrary, SR-A deficient mice were found to survive longer from pulmonary tuberculosis than the wild type [197]. Furthermore, SR-A interacts with cytoplasmic interferon-regulatory factor 5 (IRF5) and inhibits its translocation to the nucleus in Mtb infected mice leading to a shift in cytokine production from IL-12 to IL-4 and resultant suppression in Th1 response [198].

MARCO is the second member of the Mtb recognizing class A SRs (SR-A6) expressed on macrophages restricted to spleen and lymph nodes [194]. It can recognize Mtb ligand TDM and works as a co-receptor of TLR2 in activating NF- κB and eventually augments the secretion of IL-1β, IL-6, and TNF-α (Table 1) [9]. Benard and colleagues also showed similar results in the zebrafish macrophage model further extending their role in phagocytosis [199]. In another study, MARCO was found to be responsible for phagocytosis of Mtb by mesenchymal stem cells. This ligation resulted in the control of infection by autophagy and, increased production of ROS and NOS [200]. Gambian and Chinese Han populations are more susceptible when there are polymorphisms in the gene that codes for MARCO [201,202].

Scavenger receptor B1 (SR-B1) is a class B SR primarily present in liver, steroidogenic tissues as well as in macrophages. This receptor recognizes high-density lipoprotein (HDL), both oxidized and acetylated low-density lipoprotein (LDL), very-low-density lipoprotein (V-LDL), as well as anionic phospholipids [195]. SR-B1 is capable of binding Mtb, though the effects seem to be very minor and can be compensated by other macrophage receptors [196]. A recent report confirms the specific ligand for SR-B1 and describes a new role of this receptor in TB pathogenesis. Mtb uses airway microfold cells as a route to enter our body and cause infection. Microfold cell expresses a number of PRR including SR-B1. SR-B1 on microfold cells recognizes ESAT-6 secreted from Mtb and aids in transcytosis of Mtb across microfold cells (Table 1) [203].

CD36 is another class B SR (SR-B2) distributed within the cholesterol-rich microdomains of the membranes of monocytes, macrophages, and dendritic cells [194]. CD36 recognizes ManLAM and LM from Mtb cell wall leading to upregulation of TNF-α and suppression of NO secretion macrophage cell line upon prolonged incubation (Table 1) [204]. In addition to Mtb components, CD36 is responsible for the internalization of different endogenous ligands. CD36 mediates uptake and accumulation of oxLDL, produced from chronic TB-induced oxidative stress-mediated oxidation of LDL in macrophages, forming foam cells. These lipid-laden macrophages or foam cells support the survival of the pathogen due to defects in phagocytic and bactericidal activity [205]. The probable mechanism of this immune inefficiency is the accumulation of oxLDL in the lysosome leading to lysosomal dysfunction [206]. In addition, surfactant lipids of the alveolar epithelial lining increase CD36 expression, and CD36 mediated uptake of surfactant lipids supports Mtb growth in alveolar macrophages by decreasing TNF-α production [207]. These outcomes are in agreement with the report that show a better control of intracellular Mtb by CD36 deficient macrophage [208]. Accordingly, polymorphisms in the CD36 gene were seen to be connected with a lower risk of pulmonary tuberculosis among the Chinese Han population [202].

In addition to the above receptors, some other SRs or SR-like proteins are related to Mtb. One such receptor is CD163 belonging to I class SRs. It is distributed in monocytes and macrophages in both membrane-bound and soluble form that binds with hemoglobin-haptoglobin complex and mediates its clearance from plasma. Though the role of CD163 in TB pathogenesis is not clear, it serves as an important biomarker of disease status for TB because of its varying pro- and anti-inflammatory cytokines [209]. Studies show that an increased amount of both CD163+ cells and soluble CD163 indicates the severity of active TB and negative immunomodulation by Mtb through IL-10 [210,211]. Another one is the apoptosis inhibitor of macrophages (AIM) which is not a SR, rather a scavenger protein secreted by macrophages. This protein serves as a disease marker as AIM expression at the mRNA level increases after infection with Mtb. Though it is not involved in phagocytosis, an increase in AIM level is related to decreased bacterial load. Interestingly, Mtb challenged AIM expressing cells produce ROS, antimicrobial peptides cathelicidin and defensin 4B, and autophagy-related proteins Beclin 1 and LC3 **(**Table 1**)** [212].

### 1.5 Absent in Melanoma-2 (AIM2) receptor

Absent in melanoma-2 or AIM2 is a cytosolic receptor that can sense ssDNA of Mtb upon ESX-1 mediated phagosomal release and can initiate AIM2 inflammasome activation [108]. This pathway proceeds in the same way as the activation of NLRP3 inflammasome involving recruitment of the apoptosis-associated speck-like protein (ASC), activation of caspase-1, secretion of pro-inflammatory cytokines including IL-1β and IL-18 (Fig. 1), and inflammatory cell death [213]. In addition, AIM2 inflammasome mediates Th1 response after Mtb infection manifested by diminished IFN-γ production in AIM2 deficient mice [214]. Interestingly, AIM2 inflammasome activation is only seen in the case of nonvirulent Mtb as virulent Mtb can inhibit IFN-β and AIM2 inflammasome dependent IL-1β secretion indicating a role of IFN-β in activation of AIM2 inflammasome [215]. Though AIM2 knockdown results in diminished secretion of IL-1β and IL-18 by the macrophages, it increases autophagy-related proteins suggesting the role of AIM2 in suppressing autophagy [113]. Vitamin D deficiency is a major cause of TB susceptibility and reports suggest that vitamin D promotes both NLRP3 and AIM2 inflammasome activation leading to the secretion of IL-1β and anti-mycobacterial peptides [216,217]. Like AIM2, ssDNA of Mtb is sensed through cytosolic surveillance pathway (CSP) upon phagosomal escape by ESX-1. This leads to a downstream signal activating the stimulator of IFN genes (STING). Activated STING translocates to Golgi apparatus from ER as well as activates tank-binding kinase-1 (TBK) that results in activation of transcription factor IRF3 and following IFN-β production. Researchers showed that this virulence mechanism is augmented in absence of AIM2 inflammasome activation outlining the importance of AIM2 receptor in anti-Mtb immunity [218].

### 1.6 Aryl hydrocarbon receptor (AhR)

AhR is commonly known as a ligand-activated transcription factor which acts as a sensor to detect any change in cellular vicinity like oxygen levels, circadian rhythm, and redox potential, and governs the adaptation process by modulating biological approaches related to homeostasis as well as pathogenesis like inflammation. Generally, AhR gets activated by environmental toxins such as halogenated and non-halogenated polycyclic aromatic hydrocarbons, and initiates downstream signaling leading to enzymatic degradation of ligands to metabolites with diminished activity. Being widely distributed in immune cells, AhR has a significant contribution in regulating both innate as well as adaptive immunity and autoimmune reactions [219]. Moura-Alves and colleagues reported that pigmented virulence factor naphthoquinone phthiocol from Mtb binds with AhR because of its molecular resemblance to 2,3,7,8-tetrachlorodibenzo-p-dioxin (TCDD), the most common ligand of AhR. They noticed that AhR-deficient mice had higher bacterial loads in their lungs. Decreased amount of TNF-α was seen in AhR deficient lung homogenates. Though uptake of Mtb by AhR deficient bone marrow-derived macrophages (BMDMs) was unaffected, spontaneous Mtb growth and impaired production of pro-inflammatory cytokines IL-6, IL-12, as well as TNF-α were seen **(**Table 1**)** [11]. In another study, inhibition of AhR with anti-tubercular drugs resulted in impaired host defense mechanism mainly phagocytotic uptake by macrophage [220]. These observations clearly mark the anti-Mtb role of AhR.

### 1.7 CD14

Though first identified as a monocyte marker, CD14 functions as an important PRR being highly expressed on myeloid cell lineage. It was the first discovered receptor to recognize bacterial LPS and besides LPS, it can recognize a number of bacterial PAMPs including peptidoglycan and heat shock proteins. Soluble CD14 can interact with lipoarabinomannan to mount an inflammatory response. Ligand recognition by CD14 serves significant influence in MyD88 dependent inflammatory response, tumor, metabolic diseases, and atherosclerosis [221]. CD14 acts as a co-receptor in TLR2 mediated macrophage activation [222]. Furthermore, CD14 mediates endocytosis of LPS activated TLR4 and IRF3 mediated IFN-β production in early endosome [223]. The first interaction of CD14 with Mtb was identified through its ability to internalize the nonopsonized bacteria [224]. Later, it was discovered that CD14 recognizes Mtb heat shock protein Chaperonin 60.1 and stimulates the secretion of pro-inflammatory cytokines IL-1β, TNF-α, IL-6, IL-8, as well as IL-12 **(**Table 1**)** [225]. CD14 functions as a co-receptor along with MARCO and TLR2 in TDM induced inflammatory reactions [9]. Furthermore, a decrease in CD14 expression resulted in impaired T cell activation in Mtb infected diabetic mice [226]. On the contrary, Wieland and colleagues reported that CD14 confers to increased pulmonary inflammation and mortality rates [227]. However, polymorphism in the CD14 gene acts as a risk factor for developing TB [155,228]. Apart from the immunogenic role, soluble CD14 is shown to be a promising and highly accurate biomarker for TB disease [229].

### 1.8 Recent advances in immunorecognition of *Mycobacterium tuberculosis*

Some specific works have been done in recent years revealing some novel facts regarding the immune recognition of Mtb and the subsequent signaling cascades. Two novel Mtb proteins, namely Rv2659c and Rv1738 have been identified recently by Saelee et al. that can mount classical TLR-2/TLR-4 mediated immune response in human peripheral blood mononuclear cell increasing the production of IL-1β, TNF-α, IL-6, IL-8, and IL-10 [230]. Bhatt et al. revealed another two Mtb proteins, namely Rv2627c and Rv2628 as TLR agonists. They showed that these two proteins can augment the TLR-2/TLR-4 mediated downstream signaling leading to the production of proinflammatory cytokines [231]. Bomfim and colleague have extrapolated the TLR2/MyD88/NF-κB and ESX-1/STING/IFN pathway that involves induced expression of Irg1 gene and Type I-IFN production upon phagocytosis [232]. A report published by Wu and colleagues revealed the role of triggering receptor expressed on myeloid cells 2 (TREM-2) in containing Mtb. This cell membrance bound receptor is expressed on the innate immune cells of myeloid lineage that serves as an amplifier of inflammatory response in pathogenic infections. Though several ligands for this receptor has been reported, no specific Mtb ligand has been identified yet. Unlike the canonical signaling pathway observed is myeloid cells, TREM-2 can initiate inflammatory response upon ligation with unknown Mtb ligand that proceeds through STAT1/STAT4 activation and T-bet transcription leading to an enhanced proinflammatory Th1 response [233]. Naqvi and colleagues discovered another receptor belongoing to C-type lectin receptor (CLR) family, namely macrophage galactose-type lectin (MGL)-1, with novel immunomodulatory role upon recognizing Mtb. They observed marked increase in MGL-1 expression upon Mtb infection, and impaired antimycobacterial activity in macrophages and lung of MGL-1 deficient mice contributed to increased production of several anti-inflammatory cytokines including IL-1β, IL-4, IL-10, IL-13, and IFN-γ. They also observed increased expression of STAT3 protein in MGL-1 deficient mice leading to diminished T-cell mediated control of Mtb [234]. This group of researchers have made some ground breaking postulations regarding MGL, though detailed information in this regard is yet to be revealed. They have noticed that MGL receptors are activated by TLR agonists and mycobacterial lipids pointing out the overlapping role of MGL with TLRs [235]. In another study, they found that MGL silencing can lead to increased Mtb replication in human macrophages and MGL expression can be suppressed by HIV increasing patient susceptibility to Mtb infected with HIV [236]. Another novel pattern recognition receptor, called retinoic acid inducible gene I (Rig-I), has been identified to have immunostimulatory effect against Mtb. It is a cytosolic receptor that has been previously described for its role in sensing viral RNAs. This receptor interacts with mitochondrial antiviral-signaling protein (MAVS), a signal transducing adaptor protein which leads to the activation and translocation of interferon regulatory factor 7 (Irf7) and ultimately transcription of IFN-β. The report shows that Mtb SecA2 protein secretion system that contributes to the release of mycobacterial RNAs can result in Rig-I/MAVS dependant IFN- β production similar to that of viral RNAs [237]. In recent years, another group of proteins, namely Galectins have emrged as pattern recognition receptors for Mtb ligands. Galectins or β-galactoside-binding proteins are comparatively newer group of PRRs whose activities were previously limited to embryogenesis and development in mammals, but currently they are being extensively investigated for their immunomodulatory functions [238]. A number of Galectins, namely Galectin-3, -8, and -9 have been implicated to the immunological clearance of Mtb. Morrison and colleagues reported that Galectin-9 is recruited to Mtb in a ESX-1 dependent manner and binds to the surface of Mtb though they did not specify any Mtb ligand. They showed that Galectin-3, -8 and -9 triple knockout mice lacked Mtb lysogomal trafficking and autophagy. They found impaired host resistant in the knockout mice in case of chronic Mtb infection but no anomaly was orserved for chronic infection [239]. Another report shows that Galectin-8 can sense Mtb ESX-1 mediated phagosomal damage and interacts with selective autophagy adapter TAX1BP1 to target Mtb for selective autophagy [240]. A contradictory role of Galectin-9 was reported by Wu et al. showing the interaction of arabinogalactan, a Mtb virulance factor with Galectin-9 that exacerbates lung injury [241].

## 2 Conclusion

Tuberculosis has been recognized worldwide as one of the most common causes of death. To understand this disease, first, it is needed to understand its behaviors with the host cells. The interaction between *M. tuberculosis* and host immune cells is a complex story to tell. A variety of recognition receptors, both membrane-bound and cytosolic, recognize a wide repertoire of chemical structures expressed or secreted by Mtb that ranges from carbohydrates, proteins, as well as lipids to nucleic acids. Recognition of each ligand results to the activation of a distinct signaling cascade. As time is passing by, researchers are providing more updated informations about the interactions between host and Mtb. In this review, we discussed each class of pattern recognition receptors interacting with Mtb and their role in mounting protective response. We accumulated all the updated information related to these interactions and resultant signaling. Though we discussed separate pathways, but in the real world, Mtb interacts with several receptors of immune cells simultaneously. In addition to pathogen eradication, some of these interactions lead to disease persistence. Mtb has developed several virulence factors that interacts with the innate receptors to evade the protective response of host immune system. Thus, the adjustment between inflammatory as well as anti-inflammatory immune responses determines the disease status. Furthermore, knowledge of these host-Mtb interactions can not only uncover unknown aspects of immunobiology but also can be instrumental to design new therapeutic strategies. Boosting the immune response as well as bloking the immune evasion can be a promising therapeutic strategy to cure tuberculosis in this era of antibiotic resistance.

## Funding statement

No funding from any public, commercial, or not-for-profit sectors was received for this study.

## Declaration of competing interest

The authors declare that they have no known competing financial interests or personal relationships that could have appeared to influence the work reported in this paper.

## List of Abbreviations

Mtb: *Mycobacterium tuberculosis*
TB: Tuberculosis
PAMP: Pathogen-associated molecular patterns
NLR: NOD-like receptor
PRR: Pattern recognition receptors
CLR: C-type lectine receptors
TLR: Toll-like receptor
NOD: Nucleotide-binding oligomerization domain
CR: Complement receptor
SR: Scavenger receptor
AIM2: Absent in melanoma receptor-2
AhR: Aryl hydrocarbon receptor
ManLAM: Mannose capped lipoarabinomannan
LM: Lipomannan
TIRAP-Mal: TIR domain-containing adaptor
PIM: Phosphatidylinositol mannoside
MyD88: Myeloid differentiation primary response protein 88
IL: Interleukin; IRAK: IL-1 receptor-associated kinases
TRAF-6: TNF receptor-associated factor-6
LAM: Lipoarabinomannan
TAK1: TGF-β activated protein kinase-1 (TAK1)
TAB: TAK1 binding protein
NF-κB: Nuclear transcription factor-κB
TNF: Tumor necrosis factor
NEMO: NF-κB essential modulator
IκB: Inhibitor of nuclear factor kappa B
IKK: Inhibitory-κB kinase
MAPK: mitogen-activated protein kinase
MKK: MAPK kinase
ERK: Extracellular signal-regulated kinase
JNK: c-Jun N-terminal kinase
AP: Transcription activator protein
IFN: Interferon
NO: Nitric oxide
PKCzeta: Protein kinase C zeta
COX: Cyclooxygenase
PGE: Prostaglandin
PI3K: Phosphatidylinositol 3-kinase
AKT: Protein kinase B
NLRP: NOD, Leucine rich Repeat and Pyrin domain containing
NLRC: NLR family CARD domain containing
CARD: N-terminal caspase activation recruitment domain
ESX-1: ESAT-6 secretion system-1
ESAT: Early secreted antigenic target 6-kDa protein
RIPK: Receptor interacting protein kinases
TRAF: TNF-receptor associated factors
LUBAC: Linear ubiquitination assembly complex (LUBAC)
NOS: NIMA-related kinase (NEK)
ITAM: Immunoreceptor tyrosine-based activation motif
ITIM: Immunoreceptor tyrosine-based inhibitory motif
Dectin-1: Dendritic cell-associated C-type lectins
Mincle: Macrophage-inducible C-type lectin
MCL: Macrophage C-type lectin
DCAR: Dendritic cell immune-activating receptor
DC-SIGN: Dendritic cell-specific ICAM-grabbing non-integrin
MR: Mannose receptor
DCIR: Dendritic cell immunoreceptor
SP: Surfactant protein
MBL: Mannose-binding lectin
syk: Spleen tyrosine kinase
BCL-10: B-cell lymphoma/leukemia 10
MALT-1: Mucosa-associated lymphoid tissue lymphoma translocation protein-1
PKCδ: Complex through protein kinase
RAF1: Raf-1 Proto-oncogene, serine/threonine kinase
Ras: Rat sarcoma virus
FcRγ: Fragment crystallizable receptor γ-chain
TDM: Trehalose-6,6′-dimycolate
TDB: Trehalose-6,6-dibehenate (TDB)
TMM: Trehalose monomycolate (TMM)
MIP-2: Macrophage inflammatory protein-2
LARG: Leukemia-associated RHO-GEF
RHOA: Ras homolog family member A
LDL: Low density lipoprotein
AIM: Apoptosis inhibitor of macrophage
STING: Stimulator of IFN genes
TBK: tank-binding kinase
ASC: Apoptosis-associated speck-like protein
TREM 2: Triggering receptor expressed on myeloid cells 2
STAT: Signal transducer and activator of transcription
MGL: Macrophage galactose-type lectin
Rig-I: Retinoic acid inducible gene I
MAVS: Mitochondrial antiviral-signaling protein
Irf7: Interferon regulatory factor 7
